# The Internet and Clinical Trials: Background, Online Resources, Examples and Issues

**DOI:** 10.2196/jmir.7.1.e5

**Published:** 2005-03-16

**Authors:** James Paul, Rachael Seib, Todd Prescott

**Affiliations:** ^2^Department of AnesthesiaFaculty of Health SciencesMcMaster UniversityHamilton, ONCanada; ^1^Department of AnesthesiaHamilton Health SciencesHamilton, ONCanada

**Keywords:** Clinical trials, randomized controlled trial, Internet

## Abstract

Both the Internet and clinical trials were significant developments in the latter half of the twentieth century: the Internet revolutionized global communications and the randomized controlled trial provided a means to conduct an unbiased comparison of two or more treatments. Large multicenter trials are often burdened with an extensive development time and considerable expense, as well as significant challenges in obtaining, backing up and analyzing large amounts of data. Alongside the increasing complexities of the modern clinical trial has grown the power of the Internet to improve communications, centralize and secure data as well as to distribute information. As more and more clinical trials are required to coordinate multiple trial processes in real time, centers are turning to the Internet for the tools to manage the components of a clinical trial, either in whole or in part, to produce lower costs and faster results. This paper reviews the historical development of the Internet and the randomized controlled trial, describes the Internet resources available that can be used in a clinical trial, reviews some examples of online trials and describes the advantages and disadvantages of using the Internet to conduct a clinical trial. We also extract the characteristics of the 5 largest clinical trials conducted using the Internet to date, which together enrolled over 26000 patients.

## Introduction

Both the Internet and clinical trials were significant developments in the latter half of the 20th century: the Internet revolutionized global communications and the randomized controlled trial (RCT) provided a means to conduct an unbiased comparison of two or more treatments. This paper reviews the historical development of the Internet and the randomized controlled trial, describes the Internet resources available that can be used in a clinical trial, reviews some examples of online trials and describes the advantages and disadvantages of using the Internet to conduct a clinical trial.

### Historical Aspects of the Internet and Clinical Trials

#### Origins of the Internet

The Internet was born in the 1960s and its applications were initially limited by the military uses for which it was originally conceived. The original “Internet” consisted of a cooperative network of four university computers in the United States (Stanford Research Institute; University of California, Los Angeles [UCLA]; University of California, Santa Barbara; and University of Utah) [1]. The development of a protocol for information distribution in 1990 by Tim Berners-Lee paved the way for the emergence on the Internet of applications with broader public appeal [2]. Fifteen years after its inception, the World Wide Web has become a nearly indispensable tool in education, government, business, news media and, most important for the purposes of this paper, medicine and research [3]. Originally designed as an emergency communications network, the medium evolved from a communications tool for academics and the military to a medium used for education, government, business, news media, entertainment, medicine, and research. The Internet has grown at a phenomenal rate; with over 100 thousand domains or hosts in 1993 it currently has over 250 million [4]. It is the first unrestricted uncensored broadcast medium, and under ideal circumstances (namely, the right location, low traffic volumes and the right service provider), it can be very cost-effective, because unlike the telephone system, there is no charge for long-distance service.

#### Origins of the Randomized Controlled Trial

A clinical trial can be defined as any form of planned experiment involving patients [5]. The goal of a trial is to discover or verify the safety and effectiveness of interventions designed to promote wellness and prevent, diagnose, treat and provide prognosis information about disease [6]. The essence of a trial is comparison [7]. The comparison is between a group of patients who receivedtreatment with the intervention in question and a group of patients who receivedplacebo or another standard treatment. The modern clinical trial has evolved to include several features in order to provide reliable and valid results. A good trial addresses a specific clinical question for which there is equipoise (an uncertainty as to whether any of the treatments is to be preferred over the others). It uses a predefined patient population, a well-defined intervention in comparison with an appropriate control, predefined outcomes, and a methodology that involves getting informed consent from participants. Further, a trial involves appropriate blinding, randomization, and analysis. The inclusion of a control group, as opposed to historical data, is to ensure that any observed differences are due to the treatment under investigation and not another prognostic factor [5]. The purpose of randomization is to balance the treatment groups for both known and unknown prognostic factors such that any observed differences in outcome are more likely to be due to differences between the treatments in question [8]. Hence, randomization helps to prevent patient selection bias. The purpose of blinding (patients, investigators, and analysts) is to prevent outcome assessment bias.

**Figure 1:**
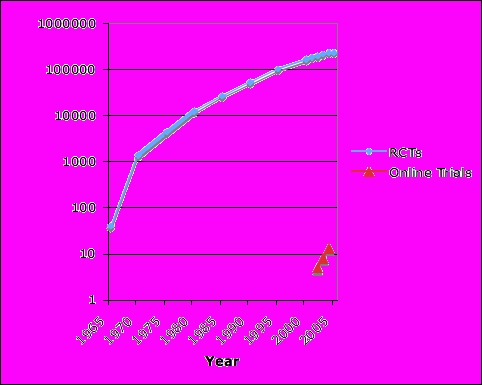
Cumulative number of randomized control trials (RCTs) versus online RCTs (based on Medline and Old Medline searches from 1950) on a logarithmic scale over time

Although many examples of clinical investigation can be found throughout the history of medicine, the RCT emerged in the mid-20th century as the most powerful and scientifically sound way to establish the efficacy and safety of medications [9]. Statistician Ronald Fisher introduced the practice of randomization (randomly assigning study participants to one or more treatment groups) in horticultural research in 1926 [6]. An epidemiologist, Austin Bradford Hill, is generally given credit for the first randomized trial involving humans in 1948 [7]. This trial, conducted by the Medical Research Council in the United Kingdom, addressed the question of whether streptomycintherapy and bed rest was more effective than bed rest alone in treating patients with pulmonary tuberculosis. In the past few decades the RCT has been increasingly used as a method to evaluate medical interventions. The Cochrane Controlled Trials Register (CCTR) is a bibliography of controlled trials generated from hand searching the world's medical journals and as of the year 2004 it identified over 415 thousand trials [10]. A recent search of the PubMed database of the National Library of Medicine in the United States yielded 65886 controlled clinical trials and 32760 of these were randomized controlled trials. This represents published trials since the mid-1960s [11]. Figure 1 illustrates the growth of RCTs. The increasing pace of RCT research is reflected by the fact that it took 21 years (1948-1969) for the first 1000 trials to be conducted yet thousands of trials were conducted in 2004 alone.

#### The Complexity of Modern Clinical Trials

An RCT is conceptually simple, but to plan a protocol for a study, obtain funding, recruit patients, conduct the trial, and analyze the data collected require considerable resources. The initial clinical trials evaluating antibiotic therapy for communicable diseases had the advantage of large treatment effects–Hill's trial on streptomycin therapy demonstrated a 74% risk reduction for mortality [12]. Today, most interventions investigated in superiority trials are expected to have a more modest benefit, perhaps a 10% to 20% risk reduction for an important outcome [6]. In order to investigate these more modest treatment effects it is necessary for modern trials to be carefully designed so that both systematic and random error are minimized, as differences of this magnitude cannot be detected reliably against a background noise of chance or other influences. Systematic error is minimized with a well-designed protocol that avoids bias, and random error is avoided by studying a large enough sample size [13]. Sample size is of particular importance in the conduct of equivalence trials. Equivalence trials, in contrast to superiority trials, are designed to establish no difference in efficacy between two interventions. However, in order to show equal efficacy, equivalence trials usually will require a 10% larger sample size in comparison with conventional superiority trials [14]. In order to achieve a sufficient sample size in a reasonable time, many trials recruit patients from multiple centers across several geographical entities (eg, cities, countries) [6]. These multicenter trials require infrastructure which is accomplished with a central coordinating center that usually handles the recruitment of study centers, the randomization of patients, any necessary laboratory analysis of patient samples, data collection, data analysis, and quality control [15].

### Internet Resources Applied to the Clinical Trial

Although the complexity of modern clinical trials is unlikely to change in the future, using Internet resources may reduce the expense and development time of a clinical trial. The Internet has many features that are useful in the conduct of a clinical trial. For instance, funding information and tools for developing a trial protocol are available online; and the processes of patient registration, randomization, data collection, analysis, and publication can all be accomplished with online resources. The Internet is also an ideal vehicle for the dissemination of information, and in this respect may facilitate the ease and rapidity with which the findings of a trial are translated into clinical practice. Table 1 summarizes a selection of Internet resources for conducting a clinical trial.

#### Online Resources for Developing a Trial Protocol

A well-designed RCT begins with the identification of a medically important question [16]. Before undertaking a new trial it is important to know what research has been done on the question in the past. To identify previous trials and systematic reviews, the Internet can be used to search online databases. Medline, EMBASE, and the Cochrane Library are online resources that can be used to quickly identify both systematic reviews and clinical trials [17]. Medline can be accessed free of charge using PubMed, but both EMBASE and the Cochrane Library require registration and an access fee [10-12]. Once relevant citations are found, most of the full text articles can be obtained by accessing the journal's home page. Members of academic institutions can often access electronic journals free of charge from their homes or offices by accessing websites via a proxy server, most often the institution's library home page [18]. Ongoing and some completed trials can be located from online trial registries in both the United States and Europe [19,20]. Online searches are useful in identifying published studies but researchers interested in exhaustive searches on a subject will have to supplement them with conventional hand searching of relevant article reference lists and by contacting experts in the area [21].

Once a research question is formulated and the literature in the field is reviewed, the Internet has tools to aid with the task of protocol development. The US National Cancer Institute maintains a website that has suggested templates for phase I – III studies, guidelines for dealing with various patient groups, as well as guidelines for formulating informed consent documents [22]. The University of California, San Francisco, School of Medicine maintains a website devoted to clinical research tools [23]. The site includes templates for study subject screening and data collection, data and safety monitoring, financial tracking, study budget, and checklists for protocol feasibility and study management. If the local expertise is not available to help with the development of the trial protocol, companies advertising online offer experienced teams of medical experts, biostatisticians, and clinical research specialists to help clients design clinical trials [24]. One of the key steps in the generation of a trial protocol is calculating the required sample size; online tools exist to perform this calculation [25].

#### Online Funding Information

A difficult hurdle is obtaining funding to conducta clinical trial. The Canadian Institute for Health Research, the National Institutes of Health in the United States, and the Medical Research Council in the United Kingdom maintain websites that contain advice to applicants and online submission forms for specific grants [26-28].

#### Study Website and Communication Amongst Trial Personnel

There are many reasons for a multicenter clinical trial to have a website [29]. A study website can be used for the following tasks: providing information to potential participants, study subjects, and investigators; listing contact information; and centralizing data handling for patient registration, randomization and data collection. Detailed information about the trial can be displayed, and the entire protocol (apart from any confidential aspects) can be made available. A secure (password protected) section of the website can be used as a powerful means of communication for trial personnel (investigators, monitors, sponsors and committee members). Today electronic mail is the standard for communication amongst members of a trial group; it is faster than conventional mail, cheaper than using long-distance telephone service, and provides an archive record of the communications. A directory on the website of the investigators, committees, sponsors, and monitors with their email addresses can help improve communications. A directory of participating centers and regional coordinators would also be helpful. A news section of the website can provide a progress report concerning the trial status and advertise upcoming meetings. A ”Frequently Asked Questions” section can provide investigators with answers to common questions regarding the study protocol, and a download page can be a means of distributing study materials (protocol, case report forms, informed consent forms) to participating study centers.

**Table 1:** Summary of Internet resources for clinical trials

**Organization**	**Universal Resource Locator (URL)**
**Funding Information**	
Canadian Institutes of Health Research	http://www.cihr.gc.ca
US National Institutes of Health	http://grants1.nih.gov/grants/index.cfm
UK Medical Research Council	http://www.mrc.ac.uk/index/funding.htm
Bibliographic Databases	
National Library of Medicine - Medline	http://www.ncbi.nlm.nih.gov/entrez/query.fcgi
The Cochrane Collaboration – The Cochrane Library	http://www.cochranelibrary.com
Elsevier Science – Bibliographic Databases	http://www.embase.com
**Clinical Trial Registries**	
National Institutes of Health – ClinicalTrials.gov	http://www.clinicaltrials.gov
Current Controlled Trials - *meta*Register of Controlled Trials	http://www.controlled-trials.com
Veritas Medicine Inc.	http://www.veritasmedicine.com
Centerwatch Clinical Trials Listing Service	http://www.centerwatch.com
**Internet Randomization Services**	
Directory of Randomization Services	http://www.sghms.ac.uk/depts/phs/guide/randser.htm
Randomization.com	http://www.randomization.com
Paradigm	http://telescan.nki.nl/paradigm.html
**Online Analysis and Sample Size Calculation**	
Simple Interactive Statistical Analysis – SISA	http://home.clara.net/sisa/index.htm
Statpages.net	http://members.aol.com/johnp71/javastat.html
**Online Publications**	
Free Medical Journals	http://www.freemedicaljournals.com/
Directory of Open Access Journals	http://www.doaj.org

#### Online Recruitment of Patients

The Internet also plays an increasing role for informing the general public about ongoing trials that are recruiting patients. Prior to the emergence of the Internet most patients were recruited for clinical trials through their physicians or perhaps through mass media advertising [30]. This system depends on individual physicians keeping up-to-date with a large range of clinical trials--an impossible task. The US Food and Drug Modernization Act of 1997 required the Department of Health and Human Services to establish a registry of clinical trials for both the government and the private sector [31]. As a result a new trial registry was launched and the home page banner reads “linking patients to medical research.”[19] The site was launched in February 2000 and currently contains approximately 11300 clinical studies sponsored by the National Institutes of Health, other US government agencies, and the pharmaceutical industry in over 90 countries. People who access the site can find trials by searching by disease condition or funding source. The website also provides information for people considering participating in a trial, including basic information on clinical trials. Several other commercial websites have been launched with the business idea of linking patients with clinical trials [32-34]. It is important for potential participants to be cautious because financial incentives used to recruit patients may interfere with ethical informed consent [30].

#### Online Patient Registration and Informed Consent

Once a patient indicates interest in participating in a particular trial, he is then screened for eligibility, and provided with the information necessary for informed consent and a consent form for signature. The necessary data for enrollment into the study is then collected. A study website can provide detailed information about the clinical trial presented in terms which the general public can understand. An online questionnaire canscreen for potential participants, and eligible patients, who elect to participate, canbe directed to the enrollment page and consent forms, made available for downloading from the website. This paradigm necessitates that potential participants have access to the Internet and that they be reasonably familiar with computers. To access the Internet, potential participants would require a personal computer, a Web browser, and access to the Internet via an Internet service provider [35]. Given these requirements, this method of patient recruitment could lead to selection bias. Surveys conducted on the demographics of Internet users show that the average user is young, white, employed, well-educated, with a higher social-economic status, and suburban [36]. Those who lack the resources for online access (for example, those with a disability that prevents access, or those who are socially disconnected or lack knowledge about Internet access points in the community) would be less likely to use the Internet and would therefore be underrepresented; whereas professionals working in the computer or telecommunications industries would likely be overrepresented.

Traditionally, study participants have signed consent documents by hand, but new legislation in both the United States and Canada has given legal weight to digital signatures for the purpose of facilitating electronic commerce [37,38]. A digital signature is a unique string that special software creates by applying a mathematical function and an encryption key to a message or file [39]. The unique string confirms both the file author's identity and the maintenance of the integrity of the file during its transmission. If accepted as ethical and legal for clinical trials, digital signatures would save the step of mailing hand-signed consent forms to the coordinating center. Regardless of the method used to obtain consent, it is important that the study participants are appropriately informed of the potential risks and benefits of the trial intervention, and of their rights regarding their electronic information. It is necessary to offer patients the option of not having their information handled electronically (for those that refuse)and to give them the option to request removal of their electronic information from the electronic environment [40]. In terms of informed consent, an argument couldbe made that all eligible patients should speak to a study representative (in person or on the phone) in order to ensure that the complexity of the study and confidentiality issues are clearly communicated and understood prior to proceeding with registration into the clinical trial. In-person contact with all trial participants wouldhelp with verification of the baseline data collection and help guard against people who might attempt to pose as a patient for mischievous reasons.

#### Online Randomization

The method of dividing subjects into groups is called random allocation or randomization and is necessary to ensure that any baseline differences between groups are due to chance alone [41]. This prevents selection bias and ensures validity of certain statistical tests. Several methods of randomizing have been used over the years, including coins, dice, cards, lots, spinning wheels, random number tables, and random number generators on computers. For multicenter trials a central coordinating center often serves as the randomization center and participating centers access the randomization allocation by a 24-hour phone service. As an alternative to this service, which can be expensive, there are several online randomization programs (some free of charge and some commercial) that can generate random allocations [42]. Randomization.com is a cost-free online randomization program that generates simple lists of allocations that can then be printed [43]. Paradigm is a Web-based randomization package developed by the Netherlands Cancer Institute and the UK Medical Research Council; it is free of charge and guides through studies interactively [44].

#### Online Data Collection

Remote data entry to a central database is one of the more useful promises of conducting a clinical trial using the Internet. A single-center clinical trial can have data entry decentralized by having a two-tier (client-server) network system that involvesindividual application instances (thick clients) running on remote computers connected to a central database server [45].

**Figure 2:**
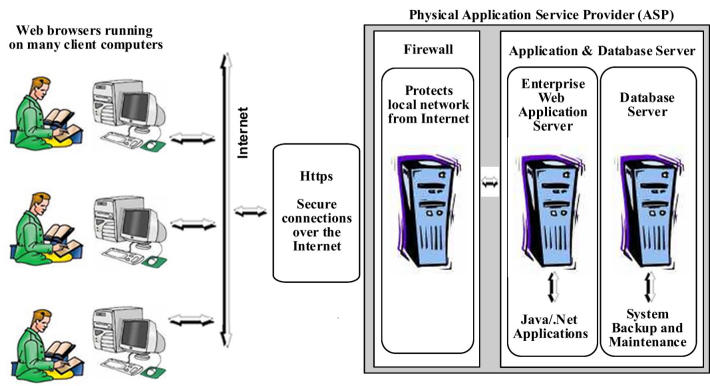
Example of a 3-tier architecture in an online clinical trial system

In a multicenter trial, participating centers can be geographically separated by great distances across several cities and countries, making the traditional local area network unfeasible. A thin-client (less bandwidth intensive) Internet-based solution canbe used to connect study centers from all over the world. An Internet data entry solution has Web-browsers - thin clients running on remote computers with the application itself running in a central Web enterprise application server. The three tiers (client/investigator, Web application server, and database server) of an online trial system are illustrated in Figure 2. With this system the following processes occur: browser requests/submits data from/to the Web application server; the Web application server in turn executes incoming business logic and submits/requests data to/from the database server; the database server saves the submitted data and sends the requested data to the Web application server; and the Web application server then executes the outgoing business logic in the application, formats the resulting data into HTML,and sends it back to the browser as a Web page. With this Web system the traditional case report forms are translated into electronic forms in HTML [46].

HTML Web pages by themselves are static text documents that cannot accept data input [47]. It is necessary to incorporate an additional enterprise application between the Web server and the database server in order to facilitate data collection, increase the efficiency of database requests and offer additional functionality for interactive real-time data validation [46]. Real-time data validation (see below) can reduce transcription errors and avoids missing data; the data thus collected should be of higher quality. The step of double keying the data for quality assurance becomes redundant [48]. This additional functionality can be incorporated by running Java or .Net code in the application server, which allows for interactive behavior with each data field [46,49]. Java is a computer programming language developed by Sun Microsystems that allows small-application programs to be downloaded from a server to a client along with the data that each program processes [50]. More commonly Java writes server-side enterprise applications that interact with Web browsers and other enterprise applications through pure HTML and Extensible Markup Language (XML) over the Internet and corporate intranet, and XML Web services (small, discrete, building-block applications that connect to each other). Microsoft .NET is a set of Microsoft software technologies for software integration through the use of XML Web services as well as to other larger applications—via the Internet [51].

XML is designed to improve the functionality of the Web by providing more flexible and adaptable information identification. It is called extensible because it is not a fixed format as is HTML (a single, predefined markup language). Instead XML is actually a metalanguage (a language for describing other languages) for designing customized markup languages for limitless types of documents [52].

#### Online Data Validation

Clinical data comes directly from the patient, the medical record, or a laboratory test. In traditional paper-based clinical trials, data is recorded on paper case report formsand then transcribed into a computer. Electronic data collection through the Internet has a number of advantages including real-time data validation, time savings due to fewer steps in data collection, and reduced handling and storage costs due to the near-elimination of paper source documents. Real-time validation could alert a researcher to an invalid entry even as he is viewing the original data source. For example, a researcher recording systolic blood pressure (in mmHg) and entering a value of 1400 couldbe prompted immediately of an invalid entry, allowing for immediate correction. The disadvantage of this electronic approach is that the US Food and Drug Administration requires validation of clinical data from each trail and it is not clear how this can be done with electronic systems. In the past the computer hardware for mobile data collection was insufficient, and study data monitors have been reluctant to embrace a fully electronic data collection model [53].

#### Online Data Analysis

After collection, data can be analyzed using online statistical tools. Simple Interactive Statistical Analysis (SISA) is one example of such Web service [25]. This Java program allows users to do statistical analysis directly on the Internet. Users select one of the procedure names, fill in a form, and click a button for immediate data analysis. Another website contains hundreds of links to free-of-charge online statistics books, tutorials, downloadable software, and related resources; immediate analysis of the results is available to the investigator [54].

#### Security Issues

Security is a central issue when considering the Internet for sensitive information exchange. Both patients and study investigators need to be confident that the data entered on electronic forms and in email communications will not be intercepted by a sniffer. A sniffer is software that monitors network traffic and it is analogous to a telephone tap [48]. The database server itself needs to be protected from intrusion from unauthorized Internet users and from unauthorized intranet users (clients connected to the local area network) [39]. Lastly, the system needs to guard against spoofing (the practice of someone pretending to be someone else) [48]. Malicious Internet users could enter fictitious patient information and invalidate the trial results. Essentially, a secure Internet clinical trial system should ensure confidentiality (information is only disclosed to users authorized to access it), integrity (information is only entered or modified by users authorized to do so), and availability (information and other resources can be accessed only by authorized users) [55].

The underlying network protocol (TCP/IP) on the Internet contains no security layer [49]. To address the issue of secure Internet transmissions, Netscape designed a nonproprietary protocol for providing data security between application protocols (such as http, telnet, NNTP, or FTP) and TCP/IP [39]. The Secure Socket Layer (SSL) provides data encryption, server authentication, and optional client authentication for a TCP/IP connection. Encrypting a file changes it from readable text to a series of numbers that only parties that have the decryption key can interpret. The latest versions of Web browsers support 128-bit encryption, translating to a code that is almost impossible to break. A computer capable of 225 million instructions per second would take a dedicated year of processor time to break such an encryption code. Documents from secure servers can be identified from the location (URL) field. The letter “s” is added to the protocol (http:// becomes https://). Encryption is also available for email communications, and this is usually (depending on the software used) can be selected as an option in the preferences menu of the email application.

After secure transmissionand storage, the data needs protection from unauthorized access once it is stored on the central database server. For this purpose firewalls – hardware and/or software that sits between the database server and the Internet – are used [39]. Further, the database server needs to be placed in a secure location so that unauthorized users cannot access it.

With the confidentiality of the clinical data maintained by encrypted transmissions and firewalls, the integrity of the data can be maintained by user logins and passwords for data entry and editing [56]. More sophisticated user authentication is possible using digital signatures. Potential spoofers have to be screened out by the enrollment procedure. This can be accomplished by communicating with the primary caregivers of potential study participants.

#### Online Publication

Currently, most major medical journals are published online and individual articles, including the title and abstract, can be browsed; full text versions are often available for download. Freemedicaljournals.com is a website that contains links to over 900 medical journals with full text articles free of charge [57]. Several mainstream journals are included but some journals limit access to articles that have been published for greater than six months to a year. Open Access journals such as those listed in the Directory of Open Access Journals (eg. BioMed Central [58] or the Journal of Medical Internet Research) offer speedy peer review and rapid publication. Article Processing Fees need to be paid from the authors' research institution or grant to cover the expenses for the peer review process and the preparation for online publication. It is the main source of income to recover publication costs for Open Access journals since the articles can be viewed free of charge and no pay-per-view charges can be imposed.

### Examples of Online Trials

Prior to the wide availability of the Internet, the The Gruppo Italiano per lo Studio della Sopravvivenza nell'Infarcto micocarico acuto (GISSI-3) Trial used telecommunications technology in the administration of the trial, [3,29]. Of the 200 participating Italian centers, 100 were provided with a computer and a modem to allow direct telephone connection to the GISSI-3coordinating center's main computer. Custom software allowed for patient enrollment, randomization, and reminder notices to the participating centers.

A Medline search of the with the search query “Internet and Clinical Trials” reveals that the Internet is increasingly being used, in whole or in part, to conduct clinical trials [40,59-73]. The 5 largest trials are summarized in Table 2. As of today, all results, except those of the ophthalmology trial which began in 2001, have been released. A large number of patients participated in Internet-based clinical trials. In these 5 trials alone over 26000 patients have been enrolled and randomized [74]. The largest Internet trial is the INVEST cardiology trial which investigated the adverse outcomes from different antihypertensive therapies. This trial alone randomized over 22000 patients. Each trial used different components of Internet technology in the administration of the studies. All 5 trials used a trial website, 4 published the protocol online, 4 allowed for online registration, 5 allowed for online data collection, and 4 used email to communicate amongst investigators. Security was approached differently as well. Two trials described using a data server firewall, 4 trials used confidential website addresses to shield their sites from spammers, 4 trials used user IDs and passwords, 3 trials described using encrypted transmissions, 2 trials did not send any patient identifying data online, and 1 trial required a 6-digit numerical code to access the website which was assigned by an RSASecurID key fob (RSA is a cryptosystem named after its inventors Rivest, Shamir and Adleman). The trials may have used other Internet technologies and security features but the preceding details are those described in their methods.

**Table 2:** Examples of clinical trials conducted using the Internet

**Number**	**Title**				**Specialty**		Year*	**References**	
**1**	Lower Pole Renal Calculi				Urology		2004	[65]	
**2**	Growth Restriction Intervention Trial (GRIT)				Obstetrics		1996	[48, 62]	
**3**	International Verapamil SR/Trandolapril Study (INVEST)				Cardiology		1997	[59,74,75]	
**4**	Osteoarthritis of the Knee: Trial of Glucosamine				Orthopedics		2000	[40]	
**5**	Intraoperative Anti-infective Prophylaxis				Ophthalmology		2001	[60]	

**Trial**		**1**	**2**	**3**		**4**			**5**
Study centers		21 centers in North America	69 centers in 13 European countries	862 centers in 14 countries		Single center			Various centers in Germany
Methodology		Multicenter randomized controlled trial	Multicenter randomized controlled trial	Multicenter randomized controlled trial		Double blind randomized controlled trial			Multicenter controlled trial
Population		Adults with lower pole renal calculi	Primary physician uncertain whether a growth restricted baby should be delivered or not	Adults with coronary artery disease and hypertension		Adult patients with osteoarthritis of the knee.			Adult patients undergoing elective cataract surgery
Sample size		112	548	22576		205			4000 to date
Intervention		Shock wave lithotripsy, percutaneous nephrolithotomy and retrograde ureteroscopic stone manipulation	Early delivery versus delayed delivery	Antihypertensive therapy with verapamil versus atenolol/hydrocholorothiazide		Glucosamine versus placebo			Irrigation with gentamicin versus regular irrigation
Outcomes		Stone removal	Perinatal mortality and developmental quotient at 2 years	Adverse outcomes: all-cause mortality, nonfatal MI, or nonfatal stroke		WOMAC pain scores			Postoperative endopthalmitis
**Internet Technologies**									
Online protocol		•†	•	•		•			
Online registration		•	•	•		•			
Online randomization		•	•	•		•			
Online data collection		•	•	•		•			•
Email communication		•		•		•			•
Data server Fifrewall		•				•			
Confidential website		•	•	•		•			•
User IDs / passwords		•	•	•		•			
Encrypted transmission		•		•					
Other		Website requires a 6-digit code assigned by an RSA SecurID key fob	No patient identifying data sent online, but by more secure means	Online ordering of study medications		Automated reminder emails and personalized schedules			No patient identifying data sent online

^*^ The year that the trial was started.

^†^ “•” Denotes that the feature was present in the trial. If the “•” is absent, the feature was not present or was not documented in the protocol.

### Advantages and Disadvantages of Online Trials

The numerous issues with online clinical trials are summarized in Table 3. Some advantages and disadvantages mentioned there are highlighted below.

#### Advantages

The main advantage of online clinical trials is the ability to centralize study information and coordinate multiple trial processes in real time at a lower cost [75]. Multicentered trials are more manageable because a system can be scaled easily to many study centers around the world without special requirements for hardware or software. The only requirement for each participating center is a computer with a Web browser and Internet access. Site training, patient recruitment, randomization, data collection, site monitoring and patient safety can all be enhanced and simplified using a clinical trial system. Further advantages include fewer personnel for trial administration, reduced or neglible paper reporting, security and backup of the entire trial at a single location, optional updating and distribution of trial protocol and data collection forms from a single location, and simplified dissemination of results.

#### Disadvantages

The key disadvantages of online trials are the real and perceived security threats that may inhibit both patients and study centers from participating. It is difficult to convince the average person of the efficiency of the abstract security measures used in Internet trials (firewalls, encrypted transmissions, password protection) compared with the conventional security measures used in traditional trials (locked file drawers). If participants are recruited through the Internet, this may lead to selection bias. Given the anonymous and transient nature of the Internet, it can be difficult for trial coordinators to assess the suitability of Internet resources that are not directly associated with well-known academic institutions. The transient and anonymous nature of the Internet is illustrated by the practice of citing the date of access for electronic resources and by the fact that many documents on the Internet do not have a documented author. If a trial relies on a third-party Internet resource, there is always the possibility that the third-party website ceases to exist prior to the completion of the study, leaving the coordinators to find an alternative resource to complete the trial. For example, finding another randomization site in the midst of a trial, which takesinto account previously allocated patients, would be problematic.

**Table 3:** Advantages and disadvantages of using the Internet to conduct clinical trials

**Topic**	**Advantages**	**Disadvantages**
Communication	• Email and website notices make exchange of information less expensive, faster and easier	• Online communications are not as secure as more traditional means (telephone, fax and mail)
Feasibility	• No need for special hardware or software at participating centers • An online clinical trial system is easier and less expensive to scale to multiple sites across multiple countries	• Risk of selection bias if all study centers are required to have Internet access
Training	• Online training resources allow for easily accessible and flexible programs for investigators	• Online training may not be as effective as a live educator
Patient recruitment	• Cost-effective broadcast medium to advertise a study to potential participants and study centers • Maintenance of a real-time view of newly registered patients	• Some patients and study centers may decline involvement because of concerns over the security of online data • May miss enrolling patients if study centers have technical difficulty with the system and do not have a study coordinator available to help them troubleshoot one-on-one
Randomization	• Eliminates the need and expense of a 24-hour call-in center for registration and randomization. • Concealment of allocation would be easier without the presence of pre-prepared randomization envelopes that have the potential to be defeated	•It is harder to locate a computer terminal than a telephone at the point of patient contact
Data collection	• Enables real-time data validation • Increased speed of data acquisition, and quality of data • Eliminates need for double-keyed data entry	• Data input could be slowed down during times of peak Internet use when access to the Web server is slowed
Monitoring	• Study monitors have real-time access to all aspects of the trial activity	• With less frequent in-person site monitoring some problems may take much longer to be identified
Safety	• Internal Review Board (IRB) has real-time access to adverse events	
Security	• Sensitive patient data is centralized in one location which simplifies security management	• Online data can be intercepted during transmission or accessed from the database server if security measures are not sufficient
Study personnel	• Fewer data entry personnel required • Fewer trial coordinators required given the centralized administration	• Requires experienced computer professionals to set up and maintain an online clinical trial system
Administration	• Reduction or elimination of paper reporting • Study protocol and data collection forms can be updated centrally and distributed to the participating centers easily • Patient data can be backed up from one location • Audit trail functionality can allow a clinical trial to be reconstructed from any point • Once a research coordinating center develops or acquires an online clinical trial system the same system could be used for multiple trials	• Because of the expense of developing an online trial system it may not be feasible for smaller trials • Would have to duplicate Internet pages in multiple languages to accommodate international trials • If a trial relies on third party Internet resources there is no guarantee that the service will remain available for the duration of the trial • Internet resources are often anonymous and transient

Other disadvantages of an online system includes system performance, lack of live support personnel, and the setup cost. The speed of the online system can be slowed significantly during peak Internet traffic and this can prolong every step of a study, from registration to data entry. The lack of a 24-hour call-in center can lead to the loss of some patients because some study centers may not be able to use online help to solve their difficulties with the study protocol or the registration and randomization steps. To set up and maintain an online clinical trial system requires experienced computer professionals. This might be too expensive for smaller trials where the administration budget is modest.

### Conclusions

Clinical trials often involve investigations of interventions of modest benefit that require multiple study centers in order to recruit a sufficient sample size in a reasonable time. The Internet can be used to administer these multicenter trials. Online resources are available to aid with each step of the study, including protocol development, identification of funding opportunities, recruitment, registration, randomization, data collection, analysis, publication and communications. The Internet has the potential to enhance clinical trials such that multicentered trials are more manageable, less expensive, easier to administer, and less time-consuming. The biggest threats to online trials are the security risks of electronic data collection, transmission, and storage. Online security measures exist but it is not clear that these are sufficient to reassure most potential study participants. We can look forward to evolving Internet technology which will bring enhanced security measures, thereby adding to the general public's comfort with electronic data.

## Abbreviations

CCTR: Cochrane Controlled Trials Register
FDA: Food and Drug Administration
HTML: Hypertext Markup Language
RCT: Randomized Controlled Trial
SISA: Simple Interactive Statistical Analysis
SSL: Secure Socket Layer
XML: Extensible Markup Language
